# Designing Carbon Nanotube-Based Oil Absorbing Membranes from Gamma Irradiated and Electrospun Polystyrene Nanocomposites

**DOI:** 10.3390/ma12050709

**Published:** 2019-02-28

**Authors:** Hemalatha Parangusan, Deepalekshmi Ponnamma, Mohammad K. Hassan, Samer Adham, Mariam Al Ali Al-Maadeed

**Affiliations:** 1 Center for Advanced Materials, Qatar University, Doha 2713, Qatar; hemakavin@gmail.com (H.P.); m.alali@qu.edu.qa (M.A.A.A.-M.); 2 ConocoPhillips Global Water Sustainability Centre (GWSC), Qatar Science and Technology Park (QSTP), Doha 24750, Qatar; Samer.Adham@conocophillips.com; 3 Materials Science & Technology Program (MATS), College of Arts & Sciences, Qatar University, Doha 2713, Qatar

**Keywords:** nanocomposite, membranes, carbon nanotubes, oil/water separation, antibacterial

## Abstract

Carbon-based materials are outstanding candidates for oil spill clean-ups due to their superhydrophobicity, high surface area, chemical inertness, low density, recyclability, and selectivity. The current work deals with the fabrication of membrane oil absorbents based on carbon nanotube (CNT) reinforced polystyrene (PS) nanocomposites by electrospinning technique. The spun membranes are also irradiated with the gamma radiation to induce enough crosslinks and thus good polymer-filler interactions. The structural, morphological, and surface properties in addition to the oil/water separation efficiency were investigated by varying the concentration of CNT and the dose of γ-irradiation. Fabricated nanofiber membranes show superior hydrophobicity and selective oil absorption at 0.5 wt.% of CNT concentration. The best mechanical properties are also obtained at this particular concentration and at 15 KGy optimum γ-irradiation dosage. The gamma irradiated PS/0.5 wt.% CNT membrane also exhibits good antibacterial effects against the bacteria, *Escherichia coli*, in the form of bacterial inhibition rings around the membranes. The present study thus shows the environmental applicability of the fabricated PS/CNT membranes in treating oil-contaminated water.

## 1. Introduction

Water and energy are the base pillars for global economic, social, and cultural development [1]. Since water is the essential life element, its pollution due to oil spills and industrial effluents has turned into a global problem. The separation of oil from water resources is crucial because of the economic and environmental challenges [2]. Numerous materials with specific wettability/hydrophobicity are reported in oil/water separation/absorption such as zeolites, clays, carbon-based materials, carbon nanotube (CNT) sponges, fibers of wool, gelators, etc. [3,4,5,6]. In order to fabricate an efficient oil/water separator, the material should possess specific properties like good hydrophobicity, oil uptake, durability, recoverability, retention over time, and biodegradability [7]. Moreover, the efficiency of oil/water separators depends on their ability to separate both water-in-oil and oil-in-water emulsions with good recyclability and reusability [2].

There are many conventional methods for oil/water separation such as skimming, centrifugation, air floatation, applying an electric field, ultrasonic method, and biological treatments. However, the chances of secondary pollution and the high cost of these methods limit their efficient utilization [8]. Polymer membranes claim good flexibility, processability, and are most probably used in manipulating superhydrophobic membranes [9]. Experimental techniques of phase separation, chemical etching, vapor deposition, sol-gel process, and electrospinning fabricate porous superhydrophobic/superoleophilic polymer-based membranes [10]. Out of all these processing methods, the technique of electrospinning is versatile and efficient in manufacturing nanofibrous membranes with superior structure and composition [11,12,13]. The electrospun membranes possess good porosity, large surface area, and fine flexibility that leads to high permeate flux when the oil/water separation is complete [14]. Reports reveal the fabrication of electrospun superhydrophobic membranes from polyurethanes, polyvinyl chloride, polysulfones, polystyrene (PS), cellulose acetate etc. [15,16,17,18,19]. The superhydrophobic nature of a particular membrane often depends on the roughness of the surface and on low surface energy [20,21], and is often achieved by proper surface treatments. The surface roughness also increases with the incorporation of nanoparticles, which leads to the formation of polymer nanocomposite membranes. Among the nanomaterials, CNT is widely applied in fabricating oil/water separating membranes due to their outstanding properties such as low density, good mechanical strength, hydrophobicity and high porosity [22,23,24,25]. 

Since electromagnetic gamma rays stimulate the surface properties of a polymer and can cause chain scission/rupture or crosslinking, many nanocomposites are gamma irradiated for modifying their surface properties [26,27,28,29]. In addition to achieving high mechanical strength, such polymer composite surfaces can behave superhydrophobic/superoleophilic, therefore repelling water and triggering oil/water separation [30]. Because superhydrophobicity is an essential concept in separating oil from water, numerous studies to mimic the lotus leaf, *Nelumbo nucifera* [31], have been done by combining surface chemistry and surface roughness of various materials. 

Numerous studies are reported for the oil-adsorbing property of CNT containing PS composites. For instance, Zhang et al. fabricated a CNT film-core shell PS-gold nanoparticle assembled underwater superoleophobic membrane capable of removing oil from contaminated water [5]. Slobodian et al. also designed PS/multi-walled (MW)CNT microspheres with excellent electrorheological effects when suspended in silicon oil [25]. Though CNT reinforced PS was the subject of study for selective oil removal, the surface property influence was not discussed in the literature. This work investigated the correlation of manufacturing technique and the surface modification by gamma irradiation to the selective oil removal from oil/water mixture. PS/CNT hybrid membranes with variable CNT loadings were fabricated by an electrospinning technique, and their properties and oil/water efficiency were studied in terms of gamma irradiation dosage and the filler loading. The fabricated electrospun membranes were also tested for their antibacterial effect, with a probable good bactericidal effect by the incorporation of CNT [32]. The gravity-driven oil/water separation efficiency of the electrospun PS/CNT membrane was investigated based on the surface modification and the contact angle studies. 

## 2. Experimental Techniques

### 2.1. Materials

PS pellets of M_W_~280,000 purchased from Sigma Aldrich (St. Louis, MI, USA) were used to prepare the polymer nanocomposites. The solvent, N,N-dimethylformamide (DMF) was also obtained from Sigma Aldrich. The properties of the polar DMF used in this study were: Boiling Point, 153 °C, Viscosity η 0.920 mPa·s at 25 °C, Dielectric constant, 36.7 at 20 °C, and surface tension σ 35.0 mn/m at 20 °C. MWCNTs of diameter 30–100 nm was obtained from Nanoshell, Panchkula, India. 

### 2.2. Fabrication of PS/CNT Electrospun Membranes

**Figure 1:**
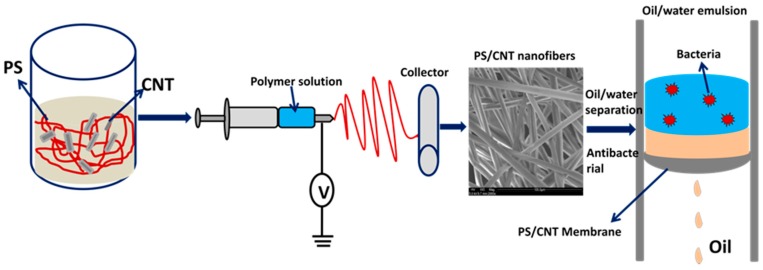
Schematic representation of the fabrication of polystyrene (PS)/carbon nanotubes (CNTs) antibacterial nanofibers and its application in oil/water separation.

PS pellets were dissolved in DMF by magnetic stirring for 3 h at 60 °C to obtain an 8% polymer solution. CNTs at different weight% (0.2%, 0.5%, and 1%) were ultrasonically dispersed in the same solvent and added to specific PS solutions. Magnetic stirring was done overnight to obtain homogeneous dispersions of PS/CNT nanocomposites. These solutions were electrospun at 1 mL/h rate using the same conditions reported earlier by our group [33]. The spun nanofibers were collected on a cylindrical rotor moving at 500 rpm, kept about 10 cm away from the needle tip at an applied voltage of 12 kV. The distance between the needle and the collector plays an important role in the morphology and fiber diameter. At a smaller needle tip to collector distance of 5 and 10 cm, defect-free fibers were obtained whereas, at longer distances of 12 and 15 cm, beads were formed on the fibers. The typical fibrous morphology of the fibers also depend on the solvent medium of dispersion and also the applied voltage. The whole procedure is schematically represented in Figure 1. Gamma irradiation was carried out at room temperature by using the Cobalt-60 with different irradiation doses (5, 10 and 15 KGy) using the instrument MDS Nordion GammaFIT^TM^ irradiation system (Nordion Inc., Ottawa, ON, Canada).

### 2.3. Characterization Methods

The morphology of the gold-coated PS/CNT samples was checked with a scanning electron microscope (FEI Nova NanoSEM 450, Thermo Fisher Scientific, Waltham, MA, USA) at 3 and 5 kV acceleration voltages. Nicolet/Fourier-transform infrared spectroscopy (FTIR) 670 (Thermo Nicolet, Thermo Fisher Scientific, Waltham, MA, USA) was used to observe the FTIR spectra of all nanocomposites. The surface properties of the samples were identified by testing the contact angle by a drop shape analysis system (OCA 35-Dataphysics, DataPhysics Instruments, Filderstadt, Germany) using deionized water. X-ray photoelectron spectroscopy (XPS, Kratos Axis Ultra DLD, Kratos Analytical Ltd., Manchester, UK) addressed the elemental composition and structural properties. Tensile properties of the polymer nanocomposites were tested using a universal testing machine (Lloyd 1KN LF Plus, AMETEK, Inc., Bognor Regis, UK) at 5 mm/min. The oil absorption capacity of PS/CNT composite membranes was monitored by keeping a specific amount of membranes in a petri dish containing engine oil/water (1:1) mixture, at room temperature. The gravity-driven test was also conducted using the same oil/water mixture by using the membrane in the funnel set up. Antibacterial activities of PS/CNT nanofibers were tested by an inhibition zone method [34], using the model bacteria, *E. coli*. Three samples, each were incubated at 37 °C for 4 h and the possibility of clear zone formation was examined. A clear zone around the sample films was recorded as an indication of *E. coli* inhibition.

## 3. Results and Discussion

### 3.1. Morphology and Wetting Performance of PS/CNT Nanofibers

**Figure 2:**
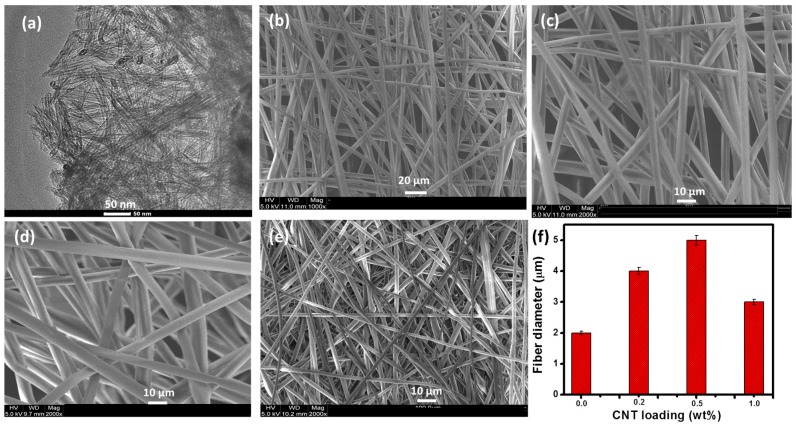
Images of (**a**) CNT and scanning electron microscope (SEM) images of (**b**) pure PS, (**c**) PS/0.2wt.% CNT, (**d**) PS/0.5wt.% CNT, (**e**) PS/1wt.% CNT, (**f**) fiber diameter distribution for all nanocomposites.

Figure 2 shows the transmission electron microscopy (CM200, Philips, Bend, OR, USA) image of MWCNT (Figure 2a) and SEM images of the pure PS and PS/CNT samples. The morphology results of the as-prepared PS and PS/CNT nanofibrous membranes, shown in Figure 2b–e, indicate the regular distribution of nanofibers from the electrospinning technique. These well distributed and randomly oriented nanofibers create a porous rough surface for the PS nanocomposite membranes.

The average diameter of the pure PS nanofiber is around 2 μm, as represented in Figure 2b. However, after CNT addition, the fiber diameter increases for the PS/CNT nanofibers as shown in Figure 2c–e. The nanofiber thickness in the case of PS/CNT composite membranes is about 3–5 μm, as shown in Figure 2c,d, which are comparable with that of pure PS. Figure 2f shows the fiber diameter distribution among all samples of PS/CNT nanocomposites. The influence of surface tension, viscosity, and conductivity of the nanocomposite solution on the fiber diameter and thus the morphology of the nanofibers is clear from the variation in fiber diameter with the CNT loading [35]. The CNTs were uniformly distributed in the PS matrix with no agglomeration, indicating its better interfacial compatibility with the polymer chains. Both the surface roughness and the surface energy of the membrane are affected by the nanofiber diameter, by enhancing the former and reducing the latter [36]. The nanofibrous nature of the membranes causes the formation of a fine, interconnected porous structure, which is highly beneficial for the oil/water separation flux [37]. Moreover, the porous nature of the electrospun membranes also depends on the application of volatile solvents during the fabrication. Such solvents encourage surface pore creation through the evaporative-cooling mechanism, in addition to the condensation of water vapor on the fiber surface [38]. In fact, other parameters such as solution viscosity, feed rates, and the applied voltage also determine the fiber diameter, fiber distribution, and therefore the porosity characteristics [39]. 

**Figure 3:**
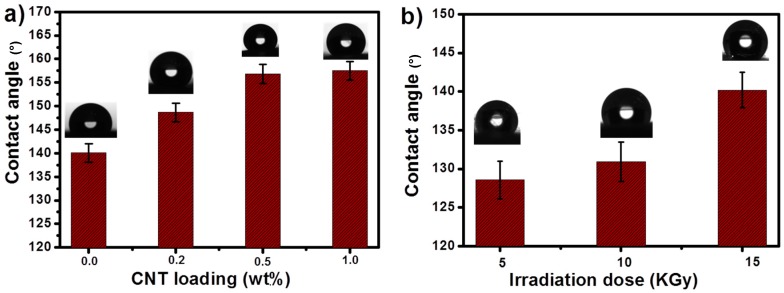
Contact angles for (**a**) pure PS and PS/CNT nanofibers, (**b**) for PS/0.5 wt.% CNT with different irradiation doses.

Since the surface energy and surface roughness depend on the contact angle values, its determination can reveal the typical surface properties of a membrane. A rough surface is significant while constructing superhydrophobic surfaces [40,41]. The obtained average contact angle values for all nanocomposites are shown in Figure 3a. From Figure 3a, it is clear that pure PS nanofibers exhibited a hydrophobic nature with a contact angle of 140°, whereas the nanocomposites, showed enhanced hydrophobicity with larger contact angle values. 

Upon CNT addition in Figure 3a, the contact angles slightly increase due to the increase in surface roughness. This results in enhanced superhydrophobicity for the composite membranes with introduced nanomaterials. Furthermore, in superhydrophobic surfaces, the chemical composition leads to lower surface free energy, and therefore surface hierarchical structures are more significant [42]. Variation in contact angle values for PS/0.5 wt.% CNT with gamma irradiation is represented in Figure 3b. The value increased from 126 to 140° depending on the different irradiation doses, but still maintaining the hydrophobicity. This substantiates the applicability of PS/CNT nanocomposite in oil/water separation.

### 3.2. Structural Characteristics of Polystyrene/CNT Nanofibers

**Figure 4:**
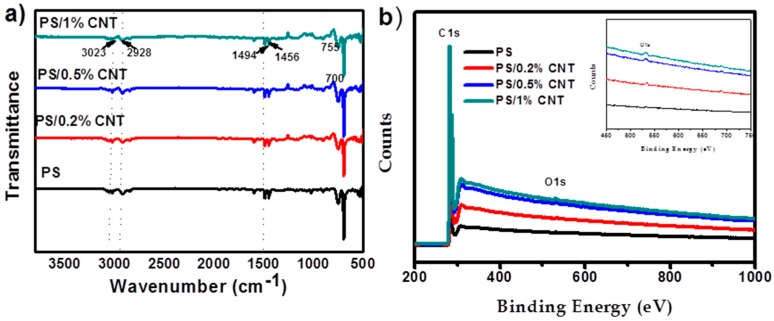
(**a**) Fourier-transform infrared spectroscopy (FTIR) spectra (**b**) X-ray photoelectron spectroscopy (XPS) spectra of pure PS and PS/CNT membrane, the inset shows the lower intense peaks related to oxygen (O1s).

Figure 4a shows the FTIR spectra of pure PS and PS/CNT nanofibers. The characteristic broad peaks at 700, 755, 1456, 1494, 2928 and 3023 cm^−1^ could be ascribed to the various absorption bands of PS [43]. The absorption intensities at 3023 and 2928 cm^−1^ correspond to the C–H symmetric and asymmetric vibrations, respectively. The peaks at 1456 and 1494 cm^−1^ were assigned to bending vibrations and the stronger peaks at 700 and 755 cm^−1^ correspond to the mono-substituted benzene. With an increase in CNT concentration, the characteristic bands of PS strengthen due to better reinforcement [44]. The absence of additional peaks indicates the absence of any chemical reaction or formation of chemical bonds during electrospinning. This illustrates the physical blending of PS and CNT. 

X-ray photoelectron spectroscopy (XPS) was used to study the surface chemical composition of the pure PS and PS/CNT membranes as represented in Figure 4b. The C1s and O1s intensities increased for the PS/CNT samples compared to the pure PS, due to the incorporation of CNTs into PS. The surface oxygen content was significantly higher with the CNT addition, as shown in the inset of Figure 4b, confirming the successful attachment of CNTs in the PS matrix. The very small peak corresponding to the oxygen comes as a result of the CNTs as the chemical vapor deposition can generate some elemental impurities on its surface. Both XPS and FTIR results indicate the existence of CNTs on the surface of PS/CNT membranes. 

### 3.3. Tensile Properties of Polystyrene/CNT Nanofibers

**Figure 5:**
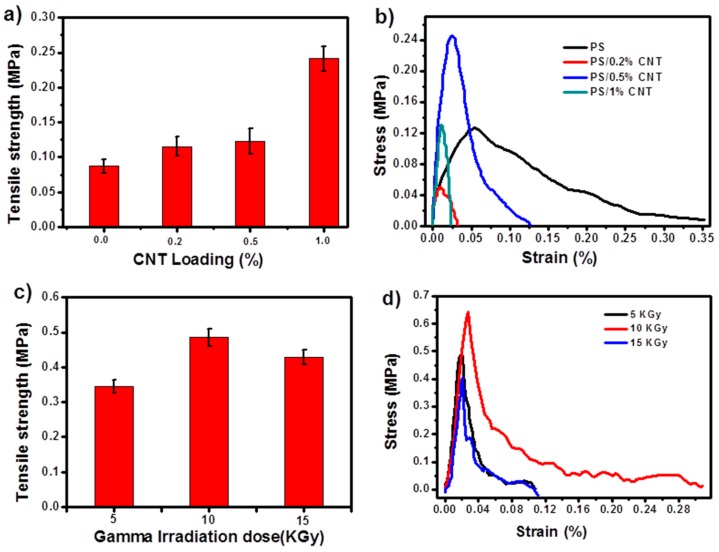
(**a**) Tensile strength and (**b**) stress-strain curve of pure PS and PS/CNT nanofibers; (**c**) tensile strength and (**d**) stress-strain curves of PS/0.5% CNT with different irradiation doses.

The tensile properties of the pure PS and PS/CNT nanofibers are shown in Figure 5, by plotting the maximum tensile strength variation and stress-strain diagram. Figure 5a shows the tensile properties of various samples containing CNTs and Figure 5c represents the tensile properties for γ-irradiated PS/0.5 wt.% CNT. Typical stress-strain curves for the non-irradiated and irradiated electrospun nanofibers are represented in Figure 5b,d respectively. The tensile properties are related to the presence of CNTs in the PS. The tensile stress for PS/CNT nanofibers at 0.5 wt.% CNT loading level was 0.24 MPa, which was higher compared to that of pure PS nanofibers (0.04 MPa). At 0.5 wt.% CNT, the cross-section gradually became denser, indicating many interactions between CNT and PS. In the electrospun membrane, the CNTs act as a crosslinking point to associate with the polymeric chain and thus increases the rigidity [45]. When CNT content further increased to 1 wt.%, the excess CNTs restrict the space for free movement of PS chains, therefore causing the mechanical properties to decrease [46]. PS/0.5 wt.% CNT irradiated nanofibers are shown in Figure 5c,d. 

The mechanical properties for PS/0.5 wt.% CNT depend on the gamma irradiation dosages. The increased properties at 10 kGy can be due to the free radicals generated on the CNT surface, which can create more interfacial bonds with the polymer chain-end groups. This crosslinking reduces chain mobility [47]. Above 15 kGy, the tensile strength began to decrease due to the possible chain scission/rupture at higher irradiation dosages.

### 3.4. Absorption and Separation of Oil and Water 

**Figure 6:**
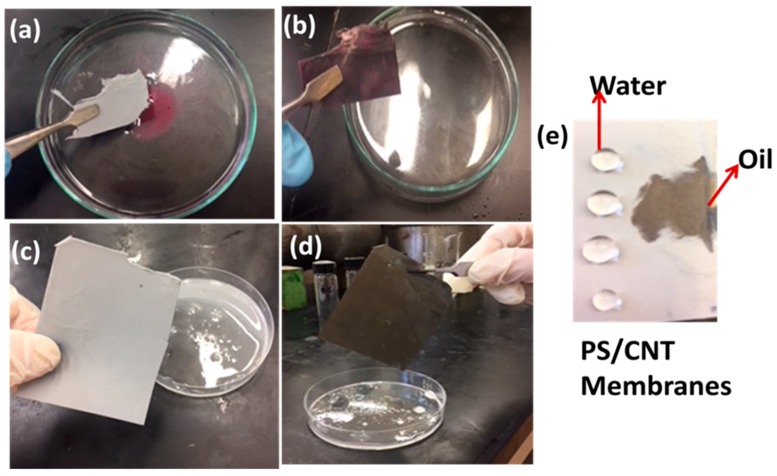
(**a**–**d**) Oil absorption capacity of dyed hydraulic oil from its mixture with water, (**e**) snapshot showing oil and water droplets on the PS/CNT membrane.

For a general understanding of the membrane reaction towards oil absorption, we selected the PS/0.5 wt.% CNT membrane irradiated with gamma ray of 15 kGy was selected for the oil/water separation test. For the experiment, hydraulic oil was selected as the model oil and it was dyed for a clear identification as shown in Figure 6a–d. The oil layer within the water was removed by a small piece of membrane, indicating the capability of the membrane for oil absorption. Figure 6e shows a snapshot of oil and water droplets on the PS/CNT nanofiber membrane. The oil droplets dropped on the surface of the PS/CNT membranes were absorbed completely and the oil contact angle was about 0°, showing superoleophilic property [22]. 

**Figure 7:**
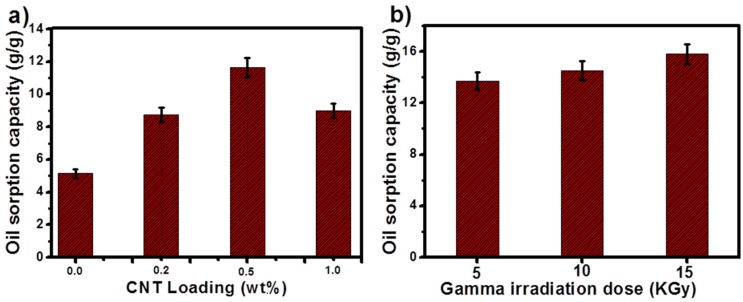
(**a**) Oil sorption capacity with different concentration of CNT (0.2, 0.5 and 1 wt.%), (**b**) with different irradiation doses.

To further evaluate the oil absorption capacity, a piece of membrane was dipped in the oil/water emulsion, as shown in Figure 6. The oil absorption capacity can be calculated by the increase in mass fraction (M: wt.%), as per the following equation,

(1) $$M=\left(m_{2}-m_{1}\right)\times m_{1}^{-1},$$

where m_1_ and m_2_ denote the weight of the membranes before and after oil adsorption [48]. In order to investigate the adsorbed oil mass, the tested membranes were taken out from the oil/water emulsion and weighed. The results are plotted in Figure 7a,b. The pure PS membrane showed low sorption capacity compared to CNT loaded membranes due to its lower porosity and the occupation of oil molecules only on the external pores. The oil sorption capacity of pure PS membrane was 5.140 g/g and the oil sorption capacity was maximum at 0.5 wt.% CNT loading with a value of 11.8 g/g. Figure 7b shows the oil sorption capacity of 0.5 wt.% CNT at different gamma irradiation doses. With the increase in irradiation dosage, the oil sorption capacity gradually increases to 16 g/g. 

The increased hydrophobicity and porosity of the membranes after gamma irradiation was due to the increase in scission of polymer chains, and the interconnected pore density of the membrane was also enhanced [49].

#### Gravity Driven Oil/Water Separation

**Figure 8:**
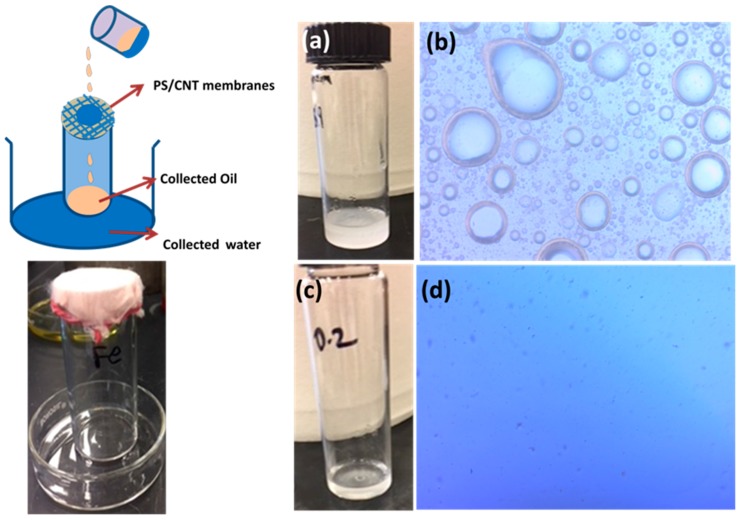
(**a**–**d**) photographs of oil/water emulsion before and after separation by the membranes (0.5 wt.% of CNT and at 15 KGy irradiated sample).

Gravity-driven oil/water separation tests were performed using the experimental set up illustrated in Figure 8. Around 20 mL oil/water emulsion was poured towards a container covered with the PS/CNT membranes at its top; the oil passed, while water retained. Separated oil was collected in the container and water was persisting on the membrane surface. This type of separation process was only driven by the weight of the oil without any external pressure and provides effective separation [19]. 

Figure 8a shows the milky white feed oil/water emulsion. The collected water and oil are shown in Figure 8c. The collected filtrate was transparent when compared with the feed emulsion. Figure 8b,d shows the optical microscopy images of feed and filtrate solutions. It can be observed from Figure 8 that the feed emulsion shows larger densely packed droplets and the droplet size is smaller for the filtrate solution, indicating the efficient separation performance for the PS/CNT membranes. 

### 3.5. Antibacterial Activity

**Figure 9:**
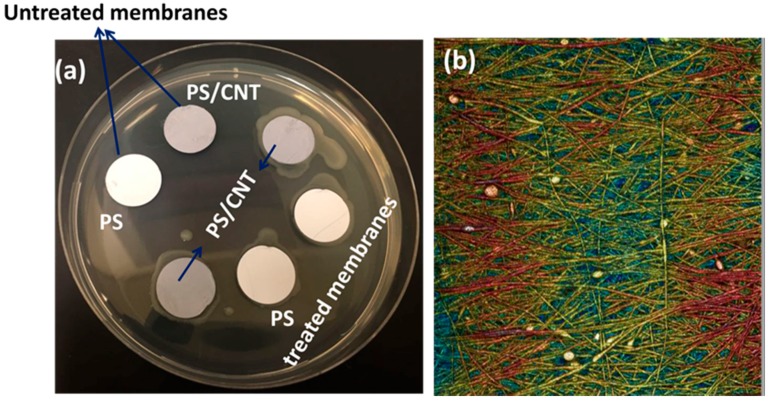
(**a**) Results of antibacterial activity on the *E. coli* bacteria, (**b**) optical microscope images of *E. coli* cells on the top of the PS/0.5 wt.% CNT and at 15 KGy gamma-ray irradiated membrane.

Water pollution causes the formation of various micro-organisms in the water, so it is important to monitor the antibacterial nature of the synthesized separating membranes. Gamma irradiated PS/0.5 wt.% CNT membrane was used for the antibacterial activity test and the selection of this membrane is done based on the characterization of the above studies. Figure 9 shows the antibacterial effect of PS/0.5 wt.% CNT and at 15 KGy gamma-ray irradiated membrane against *E. coli* and the antibacterial effect has been proven by the formation of inhibition rings around this membrane [34]. These results indicate that the PS/0.5 wt.% CNT membranes are able to eliminate *E. coli* more efficiently than the pure PS membranes. Antibacterial activity was further analyzed by the optical microscope. Figure 9b shows the optical microscope images of *E. coli* attachment onto the top of the fibers. 

## 4. Conclusions

PS/MWCNT membranes containing various concentrations of the CNT are fabricated by electrospinning and the effect of gamma irradiation on their surface characteristics are addressed. Though the nanotubes enhanced the mechanical strength of PS, the optimum concentration for enhanced oil absorbing capacity was identified as 0.5 wt.%. The hydrophobicity of the membranes, enhanced with surface roughness, gamma irradiation, and the optimum radiation dose, was 15 kGy, above which the bond rupture can probably take place. The hydrophobic/oleophobic performance of the membranes is addressed by the contact angle studies. Gravity-driven oil/water separation was clear in the case of PS/CNT at 0.5 wt.% CNT, and the oil absorption capacity (g/g) for this sample was 2.2 times higher than that of the pure polymer. Notable antibacterial properties, in terms of bacterial resistance zones, were observed in the PS/CNT irradiated at 15 kGy, indicating the fouling/bacterial degradation resistance of the sample. Thus, it is proposed that the fabricated PS/CNT membrane acts as a promising material in oil/water separation.
